# Measles second dose vaccine utilization and associated factors among children aged 24–35 months in Sub-Saharan Africa, a multi-level analysis from recent DHS surveys

**DOI:** 10.1186/s12889-022-14478-x

**Published:** 2022-11-12

**Authors:** Dagmawi Chilot, Daniel Gashaneh Belay, Kegnie Shitu, Yibeltal Yismaw Gela, Mihret Getnet, Bezawit Mulat, Atalay Goshu Muluneh, Mehari Woldemariam Merid, Desalegn Anmut Bitew, Adugnaw Zeleke Alem

**Affiliations:** 1 College of Health Sciences, Center for Innovative Drug Development and Therapeutic Trials for Africa (CDT-Africa), Addis Ababa University, Addis Ababa, Addis Ababa, Ethiopia; 2 Department of Human Physiology, College of Medicine and Health Science, School of Medicine, University of Gondar, P.O. Box 196, Gondar, Ethiopia; 3 Department of Human Anatomy, College of Medicine and Health Science, School of Medicine, University of Gondar, Gondar, Ethiopia; 4 Department of Health Education and Behavioral Science, College of Medicine and Health Science, University of Gondar, Institute of Public Health, Gondar, Ethiopia; 5 Department of Epidemiology & Biostatistics, College of Medicine and Health Science, University of Gondar, Institute of Public Health, Gondar, Ethiopia; 6 Department of Reproductive health, College of Medicine and Health Science, University of Gondar, Institute of Public Health, Gondar, Ethiopia

**Keywords:** Measles, Measles-containing vaccine doses 2, MCV2, Immunization, Vaccination, Children, Sub-Saharan Africa

## Abstract

**Background:**

Although a safe and effective vaccine is available, measles remains an important cause of mortality and morbidity among young children in Sub-Saharan Africa (SSA). The WHO and UNICEF recommended measles-containing vaccine dose 2 (MCV2) in addition to measles-containing vaccine dose 1 (MCV1) through routine services strategies. Many factors could contribute to the routine dose of MCV2 coverage remaining far below targets in many countries of this region. This study aimed to assess the prevalence of MCV2 utilization among children aged 24–35 months and analyze factors associated with it by using recent nationally representative surveys of SSA countries.

**Methods:**

Secondary data analysis was done based on recent Demographic and Health Surveys (DHS) data from eight Sub-Saharan African countries. In this region, only eight countries have a record of routine doses of measles-containing vaccine dose 2 in their DHS dataset. The multilevel binary logistic regression model was fitted to identify significantly associated factors. Variables were extracted from each of the eight country’s KR files. Adjusted Odds Ratios (AOR) with a 95% Confidence Interval (CI) and *p*-value ≤ 0.05 in the multivariable model were used to declare significant factors associated with measles-containing vaccine dose 2 utilization.

**Result:**

The pooled prevalence of MCV2 utilization in SSA was 44.77% (95% CI: 27.10–62.43%). In the multilevel analysis, mothers aged 25–34 years [AOR = 1.15,95% CI (1.05–1.26), mothers aged 35 years and above [AOR = 1.26, 95% CI (1.14–1.41)], maternal secondary education and above [AOR = 1.27, 95% CI (1.13–1.43)], not big problem to access health facilities [AOR = 1.21, 95% CI (1.12–1.31)], four and above ANC visit [AOR = 2.75, 95% CI (2.35–3.24)], PNC visit [AOR = 1.13, 95% CI (1.04–1.23)], health facility delivery [AOR = 2.24, 95% CI (2.04–2.46)], were positively associated with MCV2 utilization. In contrast, multiple twin [AOR = 0.70, 95% CI (0.53–0.95)], rural residence [AOR = 0.69, 95% CI (0.57–0.82)] and high community poverty [AOR = 0.66, 95% CI (0.54–0.80)] were found to be negatively associated with MCV2 utilization.

**Conclusions and recommendations:**

Measles-containing vaccine doses 2 utilization in Sub-Saharan Africa was relatively low. Individual-level factors and community-level factors were significantly associated with low measles-containing vaccine dose 2 utilization. The MCV2 utilization could be improved through public health intervention by targeting rural residents, children of uneducated mothers, economically poor women, and other significant factors this study revealed.

## Background

Measles is an acute respiratory illness caused by an extremely contagious virus called Morbillivirus. It is transmitted mainly through coughing and sneezing, and therefore unvaccinated individuals living nearby could be more likely to get infected [1–3]. People infected by measles develop symptoms such as high fever, cough, runny nose (coryza), red and watery eyes (conjunctivitis), and rash [4, 5]. This virus could be serious in all age groups; however, children younger than 5 years of age are more likely to suffer from measles complications. Common complications include ear infections, diarrhea, pneumonia, and encephalitis (swelling of the brain). Moreover, if acquired earlier in life, the virus could result in long-term complications of a fatal disease called subacute sclerosing panencephalitis (SSPE) [6–8].

Although a safe and effective vaccine is available since the early 1960s, measles remains an important cause of mortality and morbidity among young children globally. In 2019, about 207,500 people died and about 869,770 were infected with measles worldwide and most of them were children [9]. The African region, high in measles prevalence, is a key player in the global fight against measles [10, 11]. The strategy called Periodic Supplementary Immunization Activities (SIAs), also known as vaccination campaigns, has enhanced vaccination coverage and interrupted measles transmission in Africa [12–14]. Sub-Saharan Africa (SSA) particularly has decreased the disease substantially because of intensified measles immunization efforts. However, the heterogeneity in measles vaccination coverage across countries challenges the goal of measles elimination in SSA [15, 16].

The World Health Organization (WHO) and United Nations International Children’s Emergency Fund (UNICEF) recommended measles-containing vaccine dose 1 (MCV1) at 9 months of age, and a second dose (MCV2) of measles vaccine at age 15–18 months through routine services strategies [17, 18]. The timing for the first dose and second dose differs across countries, hence in a nation with low levels of measles transmission the first dose may be administered at 12 months and MCV2 based on programmatic considerations. However, the vaccination should not be limited to the mentioned times and every opportunity should be taken to vaccinate, particularly those < 15 years of age [19, 20].

Studies showed that the relative efficacy of two-dose (MCV1 and MCV2) is high in preventing the disease compared to only the one-dose group [21–23]. All countries have been recommended to include routine MCV2 in their national vaccination schedule regardless of the level of coverage with a routine dose of MCV1 [19]. Many countries have eradicated the virus successfully by advancing the coverage of two routine doses of the measles vaccine [24]. However, measles elimination has not been achieved due to different determinants and measles continues to be a leading cause of childhood death in developing countries [25–27].

Many factors could contribute to the routine dose of MCV2 coverage remaining far below targets in many countries of Sub-Saharan Africa. Research revealed that the socio-demographic characteristics of families as well as the communities were significant variables. In addition, knowledge, perceptions, and attitudes towards vaccination (both at the community level and individual level) had been obstacles to meeting the target of eliminating the disease. Moreover, health service availability and quality of performance, inconsistency in the opportunity of vaccination, and geographical location (residence) were important determinants for a routine dose of MCV2 coverage [28–30].

The global priorities are improving measles control, increasing awareness of vaccination, and conducting studies to answer the technical questions about measles elimination strategies [14, 31]. To control and eliminate measles in SSA, a routine dose of MCV2 coverage is a key strategy as mentioned by the WHO [19]. In addition, healthcare professionals and stakeholders should be informed of the predicted changes in measles epidemiology such as vaccine coverage, morbidity, and mortality. Moreover, previous studies were country-specific, so this study may contribute to comparing the MCV2 utilization across nations by using recent DHS surveys of countries in SSA for which the MCV2 data was available.

Given these, it is essential to expand our understanding of the current prevalence, and associated determinants. Thus the purpose of our study was to reveal the prevalence of routine dose of MCV2 utilization and analyze factors associated with it by using recent nationally representative surveys of SSA countries.

## Materials and methods

### Study design, setting, and period

Secondary analysis was performed based on the recent Demographic and Health Surveys (DHS) of eight Sub-Saharan African countries. Generally, there are thirty-six countries in the Sub-Saharan Africa region, among them only eight countries have a record of routine dose of MCV2 in their DHS dataset. Those countries were Angola, Burundi, Malawi, Nigeria, Sierra Leone, Tanzania, South Africa, and Zambia. The DHS used a cross-sectional survey study design to collect the data and the study was conducted in those eight Sub-Saharan African countries. The DHS survey in those countries was conducted from 2015 to 2019 (Table 1).

**Table 1:** Year of the survey by countries

Country	Year of survey	Country	Year of survey
Angola	2015/16	Sierra Leone	2019
Burundi	2016/17	Tanzania	2015/16
Malawi	2015/16	South Africa	2016
Nigeria	2018	Zambia	2018

### Data source

The DHS is a nationally representative survey conducted in countries with low and middle income. Eight countries’ datasets were appended together to investigate MCV2 utilization and associated factors among children aged 24–35 months in Sub-Saharan Africa. We used the Kids record dataset (KR file) and children who have data on MCV2 utilization at any time before the survey according to vaccination card, mother’s report, either vaccination card or mother’s reports were included.

**Fig. 1:**
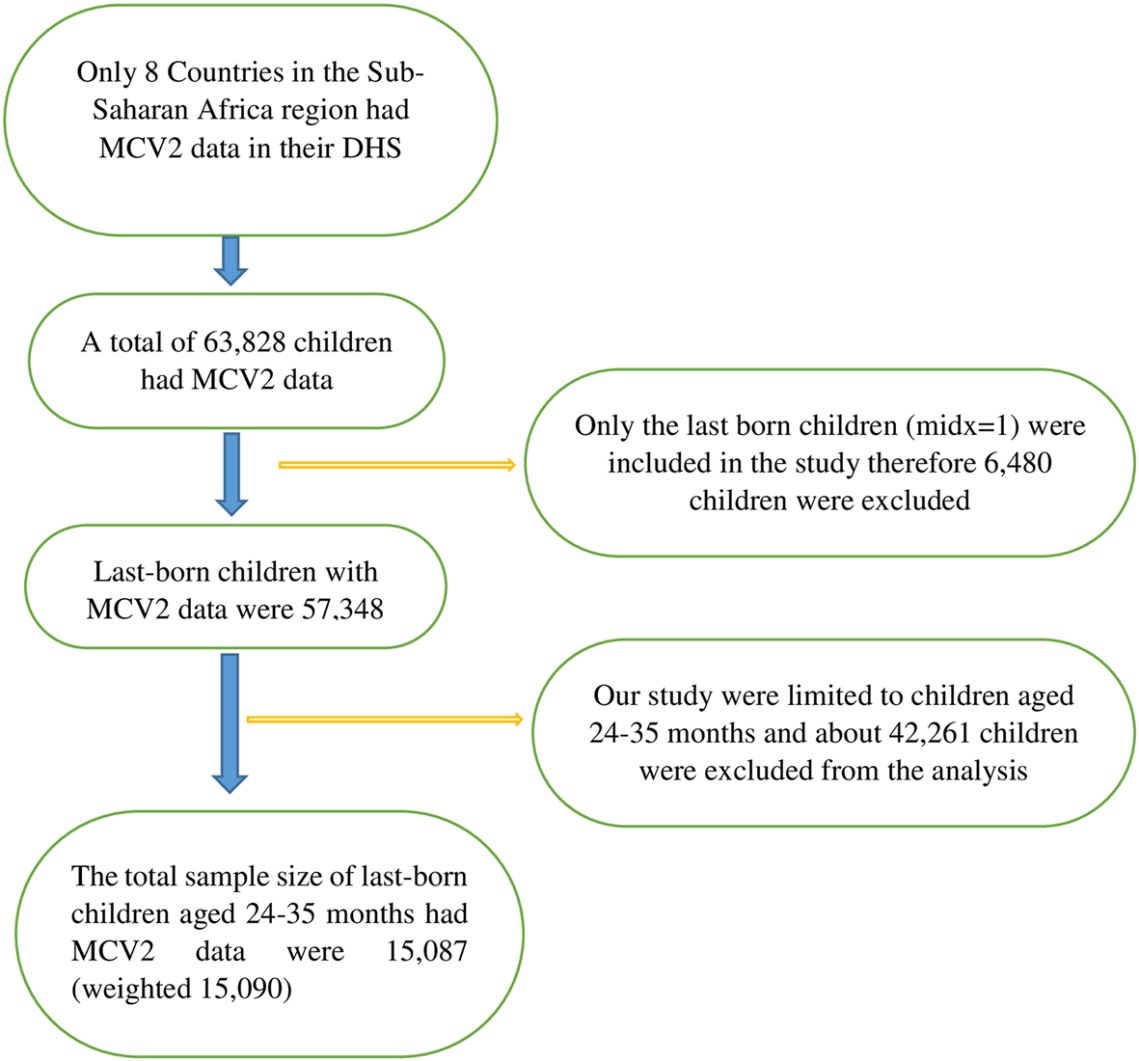
Diagrammatic representation of sample selection in the study

DHS selected the study participants by using two stages of the stratified sampling technique. In the first stage, Enumeration Areas (EAs) were randomly selected while in the second stage households were selected. In most DHS surveys the sample is selected with unequal probability to increase cases available for certain areas for which statistics are needed. We weighted the sample using the individual weight of women (v005) to produce the proper representation. Hence sample weights were generated by dividing (v005) by 1,000,000 and the total weighted sample of 15,090 children was used for the analysis (Fig. 1).

### Population

The source population for this study was children who lived in Sub-Saharan Africa. Our study population was children aged 24–35 months, who lived in Sub-Saharan Africa. The WHO recommended MCV1 to be given at 9 months of age, and MCV2 at age 15–18 months through routine services strategies [18]. The DHS data reported the percentage of children aged 12–23 months and 24–35 months who received MCV2. Therefore the age from 24 to 35 is the ideal age category for our study to get a complete record of MCV2 vaccination. We weighted (v005/1,000,000) our sample to correct over- and under-sampling and sound the findings.

### Definition of variables

#### Outcome variable

MCV2 vaccination status of children aged 24–35 months was our response variable. The outcome variable was binary and was coded as “1” if children received MCV2 and”0″ otherwise.

#### Independent variables

Independent variables were considered at two levels (individual level and community level). Individual-level (level-I) variables were maternal age, maternal education, mother’s marital status, wealth index, media exposure, sex of the child, distance to health facilities, twin status, child size at birth, sex of household head, ANC visit, PNC visits, place of delivery. Community-level (level-II) variables such as community media exposure, community women’s education, community poverty, country, and place of residence were included. We did an aggregation of individual-level variables at the cluster level and categorized them as higher or lower based on median value to generate community-level variables except for residence and country (Table 2).

**Table 2:** Independent variables of MCV2 utilization among children aged 24–35 months in SSA

Variables	Categorization / operationalization
Individual level variables	
Age of mothers	The age of mothers was categorized as 15–24, 25–34, and 35+
Mothers educational level	The mother’s educational status was categorized as uneducated, primary, secondary and above
Mothers marital status	The marital status of the mothers was categorized as married or not married.
Media access	Media access was categorized as Yes and No
Wealth index	The wealth index was categorized as Poor, Middle, and Rich.
Sex of child	The sex of the child was categorized as male or female.
Distance to a health facility	Distance to a healthcare facility was categorized as a big problem and not a big problem.
Twin status	The category for twin status was single birth and multiple births
Sex of household head	Categorized as Male and Female
ANC visit	ANC was categorized into three, No ANC visits, 1–3, and 4 + visits
PNC visit	Classified as at least one PNC visit or not having a PNC visit at all.
Place of delivery	Place of delivery was categorized as Home and Health facilities
Child size at birth	Child size at birth was categorized as large, average, and small
Community level variables	
Residence	The residents were grouped as urban, and rural
Community-level media usage	Community-level media usage was categorized as low and high. “Low” refers to communities in which < 50% of respondents had media access while “high” indicates communities in which ≥ 50% of respondents had media access.
Community-level women education	Community-level women’s education was categorized as low if communities in which < 50% of respondents had primary and above education and high if ≥ 50% of respondents had attended primary and above.
Community level poverty	Community-level poverty was categorized as low if the proportion of low wealth quintile (poorest and poorer) households was < 50% and high if the proportion was ≥ 50%.

### Statistical analyses

A multilevel binary logistic regression model was fitted to identify significantly associated factors. Variables were extracted from each of the eight country’s KR files and STATA version 14.2 was used to clean, recode and analyze the data. Pooled data were generated by appending the extracted data from the 8 Sub-Saharan African countries and weighted to draw valid inferences. Four models were applied, comprising the null model (model 0) without any explanatory variables, to test the random effect of between-cluster variability and check the existence of variation (ICC) on random intercept, Model I with individual-level variables only, to assess the impact of individual-level variables on the outcome, Model II with community-level factors only assesses the impact of community-level factors on the outcome, and Model III with both individual-level and community-level variables fitted to reveal their net fixed and random effects on the outcome variable.

Because the models were nested, we used deviance (− 2LLR) for model comparison. The intra-cluster Correlation Coefficient (ICC) was used to quantify the degree of heterogeneity of MCV2 between clusters. In addition, the Likelihood Ratio test (LR), Proportional Change in Variance (PCV), and Median Odds Ratio (MOR) were computed to measure the variation between clusters. Both community and individual-level variables with a *p*-value ≤ 0.2 in the bi-variable analysis were included in the multivariable model [32]. Adjusted OR (AOR) with 95% CI and *p* < 0.05 were applied to determine significantly associated factors. We used the variance inflation factor (VIF) test to check multicollinearity, and multicollinearity was not found because all variables have VIF < 5, and model III’s VIF was 1.49.

## Results

### Socio-demographic characteristics of respondents in SSA

In this study, 15,090 children aged 24–35 months in eight Sub-Saharan African countries were included. Of the total, 46.57% of mothers were aged 25–34 years and about one-third (31.36%) of mothers had no formal education. The majority of study participants had ANC visits (87.93) during their pregnancy, however, only 35.55% had PNC visits. About 68.87% of women deliver their children at health institutions and 66.24% were rural residents (Table 3).

**Table 3:** Socio-demographic characteristics of respondents in SSA

Variables	Categories	Unweighted frequency (%)	Weighted frequency (%)
Country	Angola	1,577 (10.45)	1,524 (10.10)
	Burundi	1,677 (11.12)	1,739 (11.53)
	Malawi	2,801 (18.57)	2,821 (18.69)
	Nigeria	4,187 (27.75)	4,239 (28.09)
	Sierra Leone	1,421 (9.42)	1,373 (9.10)
	Tanzania	1,302 (8.63)	1,293 (8.57)
	South Africa	590 (3.91)	566 (3.75)
	Zambia	1,532 (10.15)	1,533 (10.16)
Age of mothers	15–24	4,145 (27.47)	4,230 (28.03)
	25–34	6,990 (46.33)	7,028 (46.57)
	35+	3,952 (26.19)	3,832 (25.40)
Mothers educational level	No education	4,741 (31.42)	4,732 (31.36)
	Primary education	5,582 (37.00)	5,641 (37.38)
	Secondary and above	4,764 (31.58)	4,716 (31.26)
Mothers marital status	Married	10,504 (69.62)	10,597 (70.23)
	Not married	4,583 (30.38)	4,493 (29.77)
Wealth index	Poor	6,788 (44.99)	6,596 (43.71)
	Middle	3,164 (20.97)	3,039 (20.14)
	Rich	5,135 (34.04)	5,455 (36.15)
Media access	No	6,158 (40.82)	5,987 (39.67)
	Yes	8,929 (59.18)	9,103 (60.33)
Sex of child	Male	7,616 (50.48)	7,647 (50.67)
	Female	7,471 (49.52)	7,443 (49.33)
Distance to a health facility	Not big problem	5,989 (40.50)	5,928 (40.06)
	Big problem	8,800 (59.50)	8,871 (59.94)
Twin status	Single birth	14,834 (98.32)	14,841 (98.35)
	Multiple births	253 (1.68)	249 (1.65)
Sex of household head	Male	11,765 (77.98)	11,954 (79.22)
	Female	3,322 (22.02)	3,136 (20.78)
ANC visit	No	1,871 (12.40)	1,822 (12.07)
	1–3	4,446 (29.47)	4,457 (29.54)
	4+	8,770 (58.13)	8,811 (58.39)
PNC visit	No	9,734 (64.69)	9,699 (64.45)
	Yes	5,312 (35.31)	5,350 (35.55)
Place of delivery	Home	4,760 (31.55)	4,698 (31.13)
	Health facilities	10,327 (68.45)	10,392 (68.87)
Child size at birth	Large	4,804 (31.84)	4,751 (31.48)
	Average	8,335 (55.25)	8,369 (55.46)
	Small	1,948 (12.91)	1,970 (13.06)
Residence	Urban	4,805 (31.85)	5,094 (33.76)
	Rural	10,282 (68.15)	9,996 (66.24)
Community-level media usage	Low	7,592 (50.32)	7,590 (50.30)
	High	7,495 (49.68)	7,500 (49.70)
Community-level women education	Low	7,436 (49.29)	7,357 (48.75)
	High	7,651 (50.71)	7,733 (51.25)
Community poverty	Low	1,939 (12.85)	1,875 (12.42)
	High	13,148 (87.15)	13,215 (87.58)

### Prevalence of MCV2 utilization in sub-saharan african countries

**Fig. 2:**
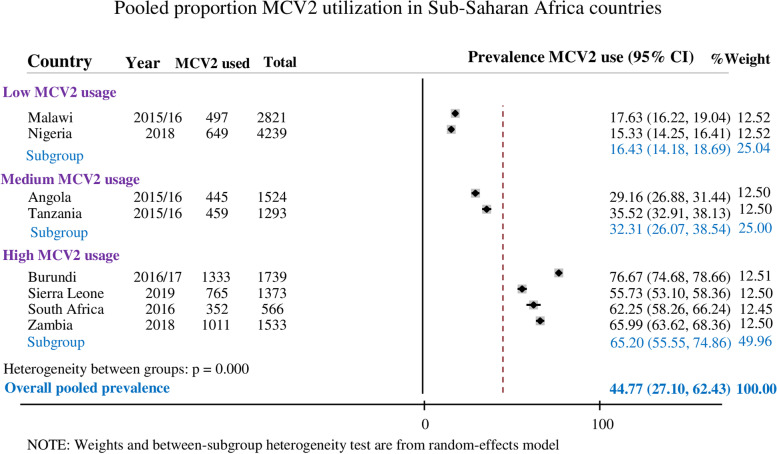
The pooled prevalence of MCV2 utilization in Sub-Saharan African countries

In eight Sub-Saharan African countries, the pooled prevalence of MCV2 utilization in children was 44.77% (95% CI: 27.10 − 62.43%). All the children had taken the MCV1 and the pooled prevalence of MCV1 utilization was 83.07% (73.51 − 92.63%). In the subgroup analysis, the prevalence of MCV2 utilization was 65.20% in countries with high MCV2 coverage while it was 16.43% in their counterparts (Fig. 2).

### Multilevel logistic regression analysis of MCV2 utilization in Sub-Saharan Africa

#### Model comparison and random effect analysis

In the random effect analysis result, the ICC value in the null model was 0.14, indicating that 14% of the total variability in MCV2 utilization was attributable to between-cluster variability, while about 86% attributable to individual differences. The MOR in the null model was 1.86, which indicates if we randomly pick a child from two separate clusters, a child with a higher probability of MCV2 vaccination in the cluster had a 1.86 times higher probability of being vaccinated than a child with lower MCV2 vaccinations in the cluster. Model III was the best-fitted model since it has the highest log likelihood (− 8912) and the lowest deviance (17,824) value. The PCV in model III was 23.8%, meaning that about 23.8% of the total variability in the MCV2 vaccination was explained by the full model (Table 3).

#### Fixed effect analysis

In the final model of multilevel logistics regression analysis; mothers aged 25–34 years, mothers aged 35 years and above, maternal had attended secondary education and above, not a big problem to access health facilities, four and above ANC visits, PNC visits, health facility delivery were positively associated variables with MCV2 utilization. Children from mothers aged 25–34 and 35 + years were 1.15 and 1.26 times more likely to utilize MCV2 than women aged 15–24 years [AOR = 1.15; 95% CI; 1.05–1.26] and [AOR = 1.26; 95% CI; 1.14–1.41] respectively. The odds of MCV2 utilization were 1.27 times higher among children from mothers whose educational status was secondary and above than mothers with no education [AOR = 1.27; 95% CI; 1.13–1.43].

The distance to health facilities was another factor. Women who responded that distance was not a big problem, their children were 1.21 times higher in MCV2 utilization compared to their counterparts [AOR = 1.21; 95% CI; 1.12–1.31]. Children from women who have attended ANC4 + and PNC visits were 2.75 and 1.13 times more likely to utilize MCV2 compared to no ANC and PNC visits [AOR = 2.75; 95% CI; 2.35–3.24] and [AOR = 1.13; 95% CI; 1.04–1.23] respectively. Children delivered in health facilities were 2.24 more likely to be vaccinated for MCV2 compared to those delivered at home [AOR = 2.24; 95% CI; 2.04–2.46].

While factors such as twins, rural residence, and high community poverty were negatively associated with MCV2 utilization. The odds of MCV2 utilization among twin children were decreased by 30% [AOR = 0.70; 95% CI; 0.53–0.95]. The odds of utilizing MCV2 among children from mothers who lived in rural was decreased by 31% compared with children whose mothers reside in urban [AOR = 0.69; 95% CI; 0.57–0.82]. High community-level poverty was also a factor for MCV2 vaccination, and children from the community with high poverty were 34% less likely to be vaccinated compared to their counterparts [AOR = 0.66; 95% CI; 0.54–0.80] (Tables 4 and 5).

**Table 4:** Multivariable multilevel logistic regression analysis results of both individual-level and community-level factors associated with MCV2 utilization in Sub-Saharan Africa

Variables	Categories	Null model	Model I AOR [95% CI]	Model II AOR [95% CI]	Model III AOR [95% CI]
Age of mothers	15–24		1.00		1.00
	25–34		1.14 (1.04—1.25)*	–––––––-	1.15 (1.05—1.26)**
	35 +		1.24 (1.11—1.37)**	–––––––-	1.26 (1.14—1.41)***
Mothers educational level	No education		1.00		1.00
	Primary Edu		0.92 (0.83—1.02)	–––––––-	0.92 (0.83—1.02)
	Secondary& above		1.23 (1.09—1.38)***	–––––––-	1.27 (1.13—1.43)***
Mothers marital status	Married		1.00	–––––––-	1.00
	Not married		0.94 (0.84—1.04)	–––––––-	0.95 (0.85—1.06)
Media access	No		1.00	–––––––-	1.00
	Yes		0.91 (0.85—0.99)*	–––––––-	0.93 (0.85—1.01)
Wealth index	Poor		1.00	–––––––-	1.00
	Middle		1.03 (0.92—1.14)	–––––––-	1.03 (0.92—1.15)
	Rich		1.04 (0.94—1.15)	–––––––-	1.08 (0.96—1.21)
Sex of child	Male		1.00	–––––––-	1.00
	Female		0.95 (0.88—1.02)	–––––––-	0.95 (0.88—1.02)
Distance to a health facility	Big problem		1.00	–––––––-	1.00
	Not big problem		1.19 (1.09—1.29)***	–––––––-	1.21 (1.12—1.31)***
Twin status	Single birth		1.00	–––––––-	1.00
	Multiple births		0.68 (0.51—0.92)*	–––––––-	0.70 (0.53—0.95)*
Sex of household head	Male		1.00	–––––––-	1.00
	Female		0.95 (0.86—1.05)	–––––––-	0.95 (0.86—1.05)
ANC visit	No		1.00	–––––––-	1.00
	1–3		1.20 (0.79—1.69)	–––––––-	1.13 (0.73 – 1.62)
	4 +		2.78 (2.36—3.27)**	–––––––-	2.75 (2.35—3.24)***
PNC visit	No		1.00	–––––––-	1.00
	Yes		1.16 (1.06—1.25)***	–––––––-	1.13 (1.04—1.23)***
Place of delivery	Home		1.00	–––––––-	1.00
	Health facilities		2.27 (2.06—2.49)***	–––––––-	2.24 (2.04—2.46)***
Child size at birth	Large		1.00	–––––––-	1.00
	Average		1.04 (0.95—1.13)	–––––––-	1.04 (0.95—1.13)
	Small		0.89 (0.78—1.01)	–––––––-	0.88 (0.78—1.01)
**Community level variables**					
Residence	Urban		–––––––-	1.00	1.00
	Rural		–––––––-	0.83 (0.76—0.90)***	0.69 (0.57—0.82)**
Community-level media usage	Low		–––––––-	1.00	1.00
	High		–––––––-	0.88 (0.78—1.00)	0.88 (0.77—1.00)
Community-level women education	Low		–––––––-	1.00	1.00
	High		–––––––-	1.06 (0.93—1.20)	0.89 (0.79—1.02)
Community level poverty	Low		–––––––-	1.00	1.00
	High			0.60 (0.49—0.72)***	0.66 (0.54—0.80)***
**Random effect**	Variance	0.52	0.51	0.49	0.42
	ICC	0.14	0.13	0.12	0.11
	MOR	1.86	1.84	1.81	1.67
	PCV	Reff	1.96	6.12	23.8
**Model comparison**	Log likelihood ratio	-9650	-8925	-9621	-8912
	Deviance	19,300	17,850	19,242	17,824
	Mean VIF		1.50	1.12	1.49

*ICC *Inter cluster corrolation cofficent, *MOR *Median odds ratio, *PCV *Proportional change in variance, *AOR *Adjusted odds ratio, *CI *Confidence intervalm, *VIF *Variance inflation factor

*P-value < 0.05

**P-value < 0.01

***P-value < 0.001

**Table 5:** Multilevel analysis of MCV2 utilization in the best Model (model III) with frequency of independent factors between vaccinated and unvaccinated as well as the COR

**Variables**	**Mcv2 utilization**		**Odds ratio**	
	No	Yes	COR **[95% CI]**	AOR **[95% CI]**
Age of mothers				
15–24	2,752 (28.73%)	1,478 (26.81%)	1.00	1.00
25–34	4,413 (46.09%)	2,614 (47.42%)	1.11 (1.02—1.22)	1.15 (1.05—1.26)**
35 +	2,411 (25.18%)	1,421 (25.77%)	1.15 (1.03—1.27)	1.26 (1.14—1.41)***
Mothers educational				
No education	3,312 (34.59%)	1,420 (25.76%)	1.00	1.00
Primary education	3,618 (37.79%)	2,022 (36.68%)	1.17 (1.06 -1.29)	0.92 (0.83—1.02)
Secondary& above	2,645 (27.62%)	2,071 (37.56%)	1.95 (1.77—2.14)	1.27 (1.13—1.43)***
Mother marital status				
Married	6,888 (71.93%)	3,709 (67.27%)	1.00	1.00
Not married	2,688 (28.07%)	1,804 (32.73%)	1.18 (1.09—1.28)	0.95 (0.85—1.06)
Media access				
No	3,935 (41.09%)	2,051 (37.21%)	1.00	1.00
Yes	5,641 (58.91%)	3,462 (62.79%)	1.11 (1.02—1.20)	0.93 (0.85—1.01)
Wealth index				
Poor	4,488 (46.87%)	2,108 (38.23%)	1.00	1.00
Middle	1,893 (19.77%)	1,146 (20.78%)	1.06 (0.99—1.19)	1.03 (0.92—1.15)
Rich	3,194 (33.36%)	2,260 (40.99%)	1.15 (1.07—1.28)	1.08 (0.96—1.21)
Sex of child				
Male	4,794 (50.07%)	2,852 (51.73%)	1.00	1.00
Female	4,781 (49.93%)	2,662 (48.27%)	0.92 (0.86—0.99)	0.95 (0.88—1.02)
Distance to a health facility				
Big problem	3,971 (41.93%)	1,957 (36.73%)	1.00	1.00
Not big problem	5,499 (58.07%)	3,371 (63.27%)	1.31 (1.21—1.41)	1.21 (1.12—1.31)***
Twin status				
Single birth	9,401 (98.18%)	5,439 (98.65%)	1.00	1.00
Multiple births	175 (1.82%)	75 (1.35%)	0.68 (0.51—0.90)	0.70 (0.53—0.95)*
Sex of household head				
Male	7,694 (80.35%)	4,259 (77.25%)	1.00	1.00
Female	1,881 (19.65%)	1,254 (22.75%)	1.07 (1.02—1.17)	0.95 (0.86—1.05)
ANC visit				
No	1,568 (16.38%)	253 (4.60%)	1.00	1.00
1–3	2,791 (29.15%)	1,665 (30.21%)	2.01 (1.58—3.03)	1.13 (0.73 – 1.62)
4 +	5,215 (54.47%)	3,594 (65.19%)	4.40 (3.79—5.11)	2.75 (2.35—3.24)***
PNC visit				
No	6,427 (67.31%)	3,271 (59.49%)	1.00	1.00
Yes	3,122 (32.69%)	2,228 (40.51%)	1.41 (1.31—1.52)	1.13 (1.04—1.23)***
Place of delivery				
Home	3,684 (38.48%)	1,013 (18.38%)	1.00	1.00
Health facilities	5,891 (61.52%)	4,500 (81.62%)	2.92 (2.67—3.18)	2.24 (2.04—2.46)***
Child size at birth				
Large	3,031 (31.66%)	1,719 (31.18%)	1.00	1.00
Average	5,226 (54.58%)	3,142 (56.99%)	1.05 (0.96—1.14)	1.04 (0.95—1.13)
Small	1,318 (13.76%)	652 (11.83%)	0.84 (0.75—0.95)	0.88 (0.78—1.01)
Residence				
Urban	3,104 (32.42%)	1,989 (36.08%)	1.00	1.00
Rural	6,471 (67.58%)	3,525 (63.92%)	0.81 (0.74—0.88)	0.69 (0.57—0.82)**
Community-level media usage				
Low	4,845 (50.60%)	2,744 (49.77%)	1.00	1.00
High	4,730 (49.40%)	2,769 (50.23%)	0.99 (0.88—1.12)	0.88 (0.77—1.00)
Community-level women education				
Low	4,818 (50.32%)	2,538 (46.04%)	1.00	1.00
High	4,757 (49.68%)	2,975 (53.96%)	1.15 (1.03—1.30)	0.89 (0.79—1.02)
Community level poverty				
Low	8,216 (85.80%)	4,998 (90.65%)	1.00	1.00
High	1,359 (14.20%)	515 (9.35%)	0.57 (0.48—0.69)	0.66 (0.54—0.80)***

*P-value < 0.05

**P-value < 0.01

***P-value < 0.001

## Discussion

Measles is a highly contagious and vaccine-preventable disease that has been a major cause of child mortality and morbidity, particularly in Sub-Saharan Africa [2, 10]. The study was aimed at quantifying MCV2 utilization and its determinants in Sub-Saharan African countries using the recent 8 countries DHS data sets. MCV2 vaccination coverage in Sub-Saharan Africa was 44.77% (95% CI: 27.10–62.43%), which significantly varied across countries ranging from 15.33% in Nigeria to 76.67% in Burundi. This variation could be because of the uneven distribution of healthcare facilities, disparities in access to immunization programs, and the attitude of populations toward the value of measles immunization [33, 34].

Individual level variables including the age of mothers, women’s education, distance to health facilities, twin status, ANC visit, PNC visit, and place of delivery were statistically associated with MCV2 utilization in the multilevel analysis. This study confirmed the association between a mother’s age and MCV2 utilization in Sub-Saharan Africa. Mothers aged 25 and above were more likely to vaccinate their children with MCV2 than mothers aged 15–24. This result was in agreement with other previous studies [32, 35]. The potential explanation for this positive relationship could be, that older mothers might be aware that lethal childhood diseases like measles can be avoided via vaccinations. In addition, aged women could have more experience in vaccinating their children and less vaccine hesitancy compared to their counterparts.

Our study showed that the odds of MCV2 utilization were higher among women who had attended secondary education and above. This is widely accepted and is consistent with the findings of several previous studies [36–39]. It might be because educated mothers may have a better understanding of how measles is fatal for their children. Besides, mothers’ level of education can positively influence practices of MCV2 utilization as they know this safe and effective vaccine prevents the disease.

Mothers who reported no problem regarding distance to access health facilities were more likely to have MCV2 utilization than those who reported it was a big problem. It is congruent with studies done in Sub-Saharan Africa and somewhere else [40–42]. This might be because of the travel cost to reach health facilities for the second dose of measles vaccination. Moreover, issues related to distance from the health facilities such as lack of suitable roads; travel phobia and motion sickness could be potential factors for missing MCV2 vaccination. The odds of MCV2 utilization among women who have twins were lower as compared to women who have single. This could be because mothers need an extra hand to take the twins to health facilities. In addition, twin mothers might have more burdens in taking care of the twins than single mothers. However, this was not found significant in other studies.

We found a strong relationship between ANC visits and MCV2 utilization among children. The odds of women who had four or more ANC visits were 2.75 times higher in MCV2 utilization of their children. This finding was supported by studies conducted in Ghana, Togo, and Ethiopia [43–45]. The possible justification could be mothers who fully attended ANC might obtain adequate counseling about the need for MCV2 utilization for these children. Consistent with previous studies [32, 46], PNC visit was found to have a significant association with MCV2 utilization in children. This could be explained by women who experienced PNC acquiring counseling and health education on the need for MCV2 for their children. In another direction, on the way to PNC, they would consistently be reminded of the schedule for MCV2 vaccination by health professionals.

The odds of women who deliver from health facilities were 2.24 times higher as compared to women who deliver at home. This finding is in agreement with other previous studies elsewhere [47, 48]. Facility delivery is the strongest factor associated with children being immunized as health professionals would tell the mothers about the importance of measles immunization. Therefore mothers who gave birth at a health facility could be more likely to comply with recommended measles immunizations for their children.

Among the community-level variables, high community poverty was significantly associated with lower odds of MCV2 utilization. This finding was in line with other research results [49–51]. Measles vaccination is provided free for children in Sub-Saharan Africa. However, childcare practices and health-seeking behavior among poorer households could be lower compared to the wealthier. Our study revealed that women who reside in rural areas were less likely to have MCV2 utilization than women living in urban. Similar results were also found in previous studies [52]. This variation between urban and rural areas could be explained by health facilities’ availability, transport accessibility, and physical proximity to health facilities.

This study has some important strengths and limitations. Among the strengths, nationally representative data from eight Sub-Saharan African countries was used. We fitted the appropriate model (multilevel analysis) to address the DHS data’s hierarchical nature. The study revealed significantly associated factors of children’s MCV2 utilization and this will have implications for policymakers and program planners to prioritize. Regarding the limitations, given the cross-sectional nature of the study design, the finding from our study may not establish a causal relationship between the individual, and community-level factors, and MCV2 utilization.

## Conclusion

With high disparity among countries, MCV2 utilization in Sub-Saharan Africa was low. Individual-level factors (younger maternal age, no maternal education, big problem accessing health facilities, twins, no ANC visit, no PNC visits, and home delivery) and community-level factors (being rural residence and high poverty) were significantly associated with low MCV2 utilization. Therefore, concerned bodies should intervene by targeting rural residents, uneducated mothers, economically poor women, and other significant factors this study revealed, to improve MCV2 utilization.

## Data Availability

Data are available online in a public, open-access repository (www.measuredhs.com/data).

## Abbreviations

AOR: Adjusted Odds Ratio
ANC: antenatal care
COR: Crude Odds Ratio
DHSs: Demographic and Health Surveys
ICC: Intra cluster correlation coefficient
KR: Kids record
MCV1: Measles Containing Vaccine dose 1
MCV2: Measles Containing Vaccine dose 2
MOR: median odds ratio
PCV: a proportional change in variance
PNC: postnatal care
SIAs: Supplementary Immunization Activities
SSA: Sub-Saharan Africa
SSPE: Subacute Sclerosing Panencephalitis
UNICEF: United Nations International Children’s Emergency Fund
WHO: World Health Organization

## References

[1] Moss WJ, Scott S. WHO immunological basis for immunization series module xx: measles. Geneva: Switzerland. 2009;5.

[2] Estofolete CF, Milhim BHGdA, CCGd F, GCDd S, Augusto MT, Terzian ACB (2020). Prevalence of measles antibodies in São José do Rio Preto, São Paulo, Brazil: a serological survey model. Sci Rep.

[3] Abbas M, Atwa M, Emara A (2007). Seroprevalence of measles, mumps, rubella and varicella among staff of a hospital in Riyadh, Saudi Arabia. J Egypt Public Health Assoc.

[4] Yanagi Y, Takeda M, Ohno S (2006). Measles virus: cellular receptors, tropism and pathogenesis. J Gen Virol.

[5] Perry RT, Halsey NA (2004). The clinical significance of measles: a review. J Infect Dis.

[6] Griffin DE (2016). The immune response in measles: virus control, clearance and protective immunity. Viruses.

[7] Mina MJ, Kula T, Leng Y, Li M, De Vries RD, Knip M (2019). Measles virus infection diminishes preexisting antibodies that offer protection from other pathogens. Science.

[8] Simons E, Ferrari M, Fricks J, Wannemuehler K, Anand A, Burton A (2012). Assessment of the 2010 global measles mortality reduction goal: results from a model of surveillance data. The Lancet.

[9] Patel MK, Goodson JL, Alexander JP, Kretsinger K, Sodha SV, Steulet C (2020). Progress toward regional measles elimination—worldwide, 2000–2019. Morb Mortal Wkly Rep.

[10] Otten M, Kezaala R, Fall A, Masresha B, Martin R, Cairns L (2005). Public-health impact of accelerated measles control in the WHO African Region 2000–03. The Lancet.

[11] Takahashi S, Metcalf CJE, Ferrari MJ, Tatem AJ, Lessler J (2017). The geography of measles vaccination in the African Great Lakes region. Nat Commun.

[12] Masresha BG, Fall A, Eshetu M, Sosler S, Alleman M, Goodson JL (2011). Measles mortality reduction and pre-elimination in the African region, 2001–2009. J Infect Dis.

[13] Organization WH (2012). Global measles and rubella strategic plan: 2012.

[14] Moss WJ, Griffin DE (2006). Global measles elimination. Nat Rev Microbiol.

[15] Goodson JL, Masresha BG, Wannemuehler K, Uzicanin A, Cochi S (2011). Changing epidemiology of measles in Africa. J Infect Dis.

[16] Strebel P, Cochi S, Grabowsky M, Bilous J, Hersh BS, Okwo-Bele JM (2003). The unfinished measles immunization agenda. J Infect Dis.

[17] Organization WH, mondiale de la Santé O (2015). Progress towards regional measles elimination, worldwide, 2000–2014. Wkly Epidemiol Record = Relevé épidémiologique hebdomadaire.

[18] Organization WH (2002). WHO-UNICEF joint statement on strategies to reduce measles mortality worldwide. Wkly Epidemiol Record = Relevé épidémiologique hebdomadaire.

[19] Organization WH (2019). Measles vaccines: WHO position paper, April 2017–Recommendations. Vaccine.

[20] Organization WH (2009). Measles vaccines: WHO position paper. Wkly Epidemiol Record = Relevé épidémiologique hebdomadaire.

[21] Garly ML, Martins CL, Balé C, da Costa F, Dias F, Whittle H (1999). Early two-dose measles vaccination schedule in Guinea-Bissau: good protection and coverage in infancy. Int J Epidemiol.

[22] Njie-Jobe J, Nyamweya S, Miles DJ, van der Sande M, Zaman S, Touray E (2012). Immunological impact of an additional early measles vaccine in Gambian children: responses to a boost at 3 years. Vaccine.

[23] Makam L, Mathew J, Ratho R, Dutta S, Singh M, Bharti B (2019). G147 Randomized controlled trial comparing anticipated measles vaccination schedules to routine vaccination starting at 9 months in indian infants. Arch Dis Child.

[24] Wolfson LJ, Grais RF, Luquero FJ, Birmingham ME, Strebel PM (2009). Estimates of measles case fatality ratios: a comprehensive review of community-based studies. Int J Epidemiol.

[25] Verguet S, Jassat W, Hedberg C, Tollman S, Jamison DT, Hofman KJ (2012). Measles control in Sub-Saharan Africa: South Africa as a case study. Vaccine.

[26] Simons E, Ferrari M, Fricks J, Wannemuehler K, Anand A (2012). .“. Assessment of the 2010 Global Measles Mortality Reduction Goal: Results from a Model of Surveillance Data”. The Lancet.

[27] Nandy R, Handzel T, Zaneidou M, Biey J, Coddy RZ, Perry R (2006). Case-fatality rate during a measles outbreak in eastern Niger in 2003. Clin Infect Dis.

[28] Geremew TT, Gezie LD, Abejie AN (2019). Geographical variation and associated factors of childhood measles vaccination in Ethiopia: a spatial and multilevel analysis. BMC Public Health.

[29] Lo Vecchio A, Cambriglia MD, Fedele MC, Basile FW, Chiatto F, del MiragliaGiudice M (2019). Determinants of low measles vaccination coverage in children living in an endemic area. Eur J Pediatrics.

[30] Mapping routine measles (2021). vaccination in low-and middle-income countries. Nature.

[31] Bester JC (2016). Measles and measles vaccination: a review. JAMA Pediatr.

[32] Tesema GA, Tessema ZT, Tamirat KS, Teshale AB (2020). Complete basic childhood vaccination and associated factors among children aged 12–23 months in East Africa: a multilevel analysis of recent demographic and health surveys. BMC Public Health.

[33] Adetokunboh O, Iwu-Jaja CJ, Nnaji CA, Ndwandwe D (2021). Missed opportunities for vaccination in Africa. Curr Opin Immunol.

[34] Jheeta M, Newell J. Childhood vaccination in Africa and Asia: the effects of parents’ knowledge and attitudes. SciELO Public Health; 2008. p. 419-A. 10.2471/BLT.07.047159 PMC2647458 18568264

[35] Oleribe O, Kumar V, Awosika-Olumo A, Taylor-Robinson SD. Individual and socioeconomic factors associated with childhood immunization coverage in Nigeria. Pan Afr Med J. 2017;26. 10.11604/pamj.2017.26.220.11453 PMC5491752 28690734

[36] Brownwright TK, Dodson ZM, van Panhuis WG (2017). Spatial clustering of measles vaccination coverage among children in sub-Saharan Africa. BMC Public Health.

[37] Abebe DS, Nielsen VO, Finnvold JE (2012). Regional inequality and vaccine uptake: a multilevel analysis of the 2007 Welfare Monitoring Survey in Malawi. BMC Public Health.

[38] Majekodunmi OB, Oladele EA, Greenwood B. Factors affecting poor measles vaccination coverage in sub-Saharan Africa with a special focus on Nigeria: a narrative review. Trans R Soc Trop Med Hygiene. 2022. 10.1093/trstmh/trac013 35294971

[39] Kamau N, Esamai F (2001). Determinants of immunization coverage among children in Mathare Valley, Nairobi. East Afr Med J.

[40] Wiysonge CS, Uthman OA, Ndumbe PM, Hussey GD (2012). Individual and contextual factors associated with low childhood immunisation coverage in sub-Saharan Africa: a multilevel analysis. PLoS ONE.

[41] Russo G, Miglietta A, Pezzotti P, Biguioh RM, Mayaka GB, Sobze MS (2015). Vaccine coverage and determinants of incomplete vaccination in children aged 12–23 months in Dschang, West Region, Cameroon: a cross-sectional survey during a polio outbreak. BMC Public Health.

[42] Okwaraji YB, Mulholland K, Schellenberg J, Andarge G, Admassu M, Edmond KM (2012). The association between travel time to health facilities and childhood vaccine coverage in rural Ethiopia. A community based cross sectional study. BMC Public Health.

[43] Landoh DE, Ouro-Kavalah F, Yaya I, Kahn A-L, Wasswa P, Lacle A (2016). Predictors of incomplete immunization coverage among one to five years old children in Togo. BMC Public Health.

[44] Abadura SA, Lerebo WT, Kulkarni U, Mekonnen ZA (2015). Individual and community level determinants of childhood full immunization in Ethiopia: a multilevel analysis. BMC Public Health.

[45] Laryea DO, Parbie EA, Frimpong E (2014). Timeliness of childhood vaccine uptake among children attending a tertiary health service facility-based immunisation clinic in Ghana. BMC Public Health.

[46] Mrisho M, Obrist B, Schellenberg JA, Haws RA, Mushi AK, Mshinda H (2009). The use of antenatal and postnatal care: perspectives and experiences of women and health care providers in rural southern Tanzania. BMC Pregnancy Childbirth.

[47] Sarker AR, Akram R, Ali N, Chowdhury ZI, Sultana M (2019). Coverage and determinants of full immunization: vaccination coverage among Senegalese children. Medicina.

[48] Mutua MK, Kimani-Murage E, Ettarh RR (2011). Childhood vaccination in informal urban settlements in Nairobi, Kenya: who gets vaccinated?. BMC Public Health.

[49] Chidiebere ODI, Uchenna E, Kenechi O (2014). Maternal sociodemographic factors that influence full child immunisation uptake in Nigeria. South Afr J Child Health.

[50] Halder AK, Kabir M (2008). Inequalities in infant immunization coverage in Bangladesh. Health Serv Insights.

[51] Singh PK, Parasuraman S (2014). ‘Looking beyond the male–female dichotomy’–sibling composition and child immunization in India, 1992–2006. Soc Sci Med.

[52] Doctor HV, Findley SE, Bairagi R, Dahiru T. Northern Nigeria maternal, newborn and child health programme: selected analyses from population-based baseline survey. Open Demography J. 2011;4(1).

